# Effect of sonic and ultrasonic activation on physicochemical properties of root canal sealers

**DOI:** 10.1590/1678-7757-2018-0556

**Published:** 2019-08-30

**Authors:** Fabiane Carneiro Lopes, Caio Zangirolami, Jardel Francisco Mazzi-Chaves, Alice Corrêa Silva-Sousa, Bruno Monguilhott Crozeta, Yara Teresinha Corrêa Silva-Sousa, Manoel Damião Sousa-Neto

**Affiliations:** 1 Universidade de São Paulo Universidade de São Paulo Faculdade de Odontologia de Ribeirão Preto Departamento de Odontologia Restauradora Ribeirão Preto São Paulo Brasil Universidade de São Paulo, Faculdade de Odontologia de Ribeirão Preto, Departamento de Odontologia Restauradora, Ribeirão Preto, São Paulo, Brasil.; 2 Universidade de Ribeirão Preto Universidade de Ribeirão Preto Faculdade de Odontologia Ribeirão Preto São Paulo Brasil Universidade de Ribeirão Preto, Faculdade de Odontologia, Ribeirão Preto, São Paulo, Brasil.

**Keywords:** Root canal filling materials, Physicochemical analysis, Ultrasonics

## Abstract

**Objective:**

To evaluate the effect of ultrasonic and sonic activation on physicochemical properties of AH Plus, MTA Fillapex, ADSeal, GuttaFlow Bioseal, and GuttaFlow 2 sealers.

**Methodology:**

Three experimental groups were formed: no activation (NA), ultrasonic activation (UA), and sonic activation (SA). The sealers were manipulated according to the manufacturers’ instructions. A 3-mL syringe was adapted to receive 1 mL of sealer. Activation was performed with a 20/.01 ultrasonic insert (20 s/1W) in the UA group. A size 35.04 sonic tip was used (20 s/10,000 cycles/min-1) in the SA group. The molds for physicochemical analysis were filled and evaluated according to ANSI/ADA specification no. 57: setting time (ST), flow (FL), dimensional change (DC), solubility (SB), and radiopacity (RD). Statistical analysis was performed by Kruskal-Wallis, one-way ANOVA, and Tukey's tests (P<0.05).

**Results:**

Regarding ST, only AH Plus and GuttaFlow 2 in the NA group met the ANSI/ADA standards. All FL values were greater than 20 mm in diameter, as determined by ANSI/ADA. The tested sealers and protocols did not comply with the ANSI/ADA standards for DC. As for SB, only MTA Fillapex, regardless of the activation protocol, did not follow the ANSI/ADA standards. All of the investigated sealers, regardless of the activation protocol, presented radiographic density higher than 3 mm Al, as proposed by ANSI/ADA.

**Conclusions:**

UA and SA promoted changes in the physicochemical properties of the evaluated root canal sealers, mainly in ST and F. Thus, it is important to evaluate the physicochemical properties of endodontic sealers associated with activation techniques prior to clinical application in order to determine whether the properties follow the parameters set by ANSI/ADA, ensuring safety and quality of root canal filling.

## Introduction

Root canal system fillings should provide adequate sealing in order to prevent percolation of fluids and reinfection, allowing for the repair of the apical and periapical regions.^1^^,^^2^ The quality of obturations is directly related to the material and the technique employed.^2^^–^^6^

Regarding root canal filling techniques, lateral condensation presents some limitations in irregular, flat, and complex root canal systems.^3^^,^^5^^–^^7^ A number of sealing techniques have been proposed over the years, including thermomechanical and thermoplastic techniques. The thermomechanical technique promotes apical and lateral condensation of the filling material against the root canal walls, favoring the homogeneous filling of irregularities and accessory canals.^8^ On the other hand, the thermoplastic technique plasticizes gutta-percha, forming a homogeneous mass compacted towards the apical region, allowing the sealing and filling of the root canal system with more homogeneous distribution of the filling material.^4^^,^^6^^,^^7^^,^^9^

Recent studies have demonstrated the presence of gaps and voids after the use of different root canal filling techniques.^1^^,^^3^^,^^4^^,^^6^ In an attempt to overcome these limitations, some authors have proposed the sonic and ultrasonic activation of sealers causing them to penetrate into the canal, promoting a better adaptation between the sealer and the root canal walls.^1^^,^^6^^,^^10^

Ultrasonic techniques are based on the use of inserts that, at high power, promote acoustic transmission and a subsequent cavitation effect, which minimizes the formation of voids inside the filling material and allows a greater adaptation of the sealers to the root canal walls and irregularities, as well as greater penetration into lateral and accessory canals.^1^^,^^6^^,^^10^^,^^11^ Sonic activation, however, allows for short movements of the tips inside the canal with low-frequency vibration, generating a hydrodynamic phenomenon that increases the penetration of the endodontic sealer in areas of difficult access.^6^^,^^10^ Nevertheless, to date, it has not been possible to state the real influence of these activation protocols on the alteration of the physicochemical properties of endodontic sealers.

Root canal sealers can be classified according to their composition: zinc oxide- and eugenol-based sealers; sealers containing calcium hydroxide; epoxy resin-based sealers; glass ionomer sealers; methacrylate resin-based sealers; or silicone- and bioceramic-based sealers.^6^^,^^12^^–^^15^ Note that, despite the wide variety, resin-based sealers are the most widely used ones, and, more recently, both silicone- and bioceramic-based sealers have been gaining attention in clinical practice.

Given the broad use of protocols for activation of root canal sealers, it is necessary to understand how they act on the physicochemical properties of different types of endodontic sealers. Thus, the objective of this study was to evaluate the physicochemical properties of setting time, flow, dimensional change, solubility and radiopacity, in accordance with ANSI/ADA specification no. 57,^12^^,^^16^ of different sealers without activation and after ultrasonic and sonic activation. The null hypothesis was that ultrasonic and sonic activation protocols would not change the physicochemical properties of the tested root canal sealers.

## Methodology

**Figure 1:**
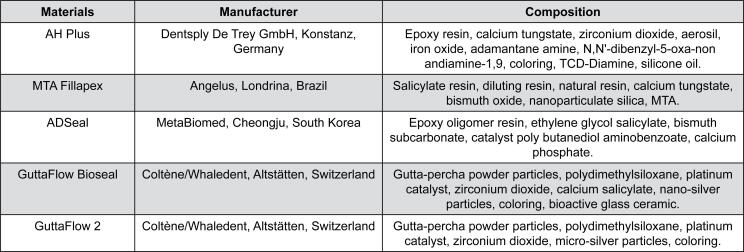
Composition of root canal sealers and their manufacturers

AH Plus (Dentsply DeTrey, Konstanz, Germany), MTA Fillapex (Angelus, Londrina, Paraná, Brazil), ADSeal (MetaBiomed, Cheongju, South Korea), GuttaFlow Bioseal (Coltene/Whaledent, Langenau, Germany), and GuttaFlow 2 (Coltene/Whaledent, Langenau, Germany) sealers were manipulated according to the manufacturers’ instructions. The tested materials were conditioned at 23±2°C for a period of at least 24 hours prior to testing, as recommended by ANSI/ADA specification no. 57. Information about the tested sealers (manufacturer and composition) is shown in Figure 1.

Three experimental groups were formed according to the sealer activation protocol.

Group I -no activation: after sealer manipulation in glass plates, the molds were filled for further analysis.

For groups II and III, the sealers were manipulated in glass plates and transferred to a 3-mL disposable plastic syringe adapted to receive 1.0 mL of sealer and activated as described below for each group. After activation, the sealers were immediately transferred from the syringe to the molds according to the analysis of each physicochemical property.

Group II – sonic activation: the sealers were activated using size 35/.04 tip of a sonic device EndoActivator^®^ (Dentsply Tulsa Dental Specialties, Tulsa, Oklahoma, USA) for 20 seconds at 10,000 cycles per minute^−1 6^ without touching the syringe walls.

Group III - ultrasonic activation: the sealers were activated with the insertion of a size 20/.01 taper (E1 - Irrisonic Tip^®^) (Helse Dental Technology, São Paulo, Brazil) and ultrasonic device (EMS, Le Sentier, Switzerland) at power level 1 for 20 seconds,^6^ without touching the syringe walls.

The setting time, flow, dimensional change, solubility, and radiopacity tests were performed following the method employed in previous studies.^13^^–^^16^

### Setting time

For the setting time test, five circular Teflon^®^ molds (10 mm in diameter and 2 mm in thickness) were fixed on glass plates and completely filled with the sealers previously prepared according to each group. After about 150±10 seconds, a 100±0.5 g Gilmore needle apparatus with a flat end of 2.0±0.1 mm in diameter was carefully lowered vertically onto the horizontal surface of each sample. The needle tip was cleaned and probing was repeated until formed indentations ceased to be visible. The time elapsed between sealer preparation until the moment when the Gilmore needle apparatus marks were no longer visible on the sealer surface was considered as the setting time. If the results differed by more than ±5%, the test was repeated. This test was performed under controlled temperature (37±1°C) and relative humidity (RH) (95±5%) following the method used by Flores, et al.^14^ (2011) and Camargo, et al.^15^ (2017).

### Flow

For the flow tests, as performed by Camargo, et al.^15^ (2017), 0.5 mL of the sealer, previously manipulated and prepared according to each group, was placed at the center of a glass plate (40 mm × 40 mm × 3 mm) with the aid of a graduated syringe. Afterwards, a second glass plate weighing 120 g was placed on top of the sample. After 10 minutes, the largest and smallest diameters of the samples were measured using a digital caliper. In this test, the difference between the larger and smaller diameters should not exceed 1 mm, and the sealers should be uniform. If the difference between the major and minor diameters exceeded 1 mm, the test was repeated according to ANSI/ADA specification No. 57.

### Dimensional change

For dimensional change assessment, five cylindrical Teflon^®^ molds measuring 3 mm in diameter and 3.58 mm in height were placed on a glass plate wrapped with a fine cellophane sheet, and filled with a slight excess of freshly prepared sealer. Then, a microscope slide, also wrapped in cellophane, was pressed onto the upper surface of the mold. The assembled group was kept firmly joined by a C-shaped clamp, transferred to an incubator (37±1°C, 95±5% RH), and left to stand for a period corresponding to three times the sealer setting time. Afterwards, the flat end of the molds containing the samples were ground with 600-grit wet sandpaper.^15^^,^^16^

The samples were removed from the mold, measured with a digital caliper, stored in a 50-mL vessel containing 2.24 mL of deionized distilled water, and kept in an incubator (37±1°C, 95±5% RH) for 30 days. The samples were then removed from the container, blotted dry with absorbent paper, and measured again for length. The percentage of the dimensional alterations was calculated using the formula: [(L30 -L) / L] × 100, where L30 is the length of the sample after 30 days of storage and L is the initial length of the sample.^15^^,^^16^

### Solubility

For the solubility tests, 10 circular Teflon molds with 7.75 mm of inner diameter and 1.5-mm in thickness were filled with freshly prepared sealer. Each mold was supported by a larger glass plate covered with a cellophane sheet. An impermeable nylon thread was placed inside the material, and another glass plate, also covered with cellophane sheet, was positioned on top of the mold and pressed manually in such a way that the plates touched the entire mold in a uniform manner. The assembly was placed in an incubator (37±1°C, 95±5% RH) and left to stand for a period corresponding to three times the sealer setting time. As soon as the samples were removed from the mold, they were weighed three times each using a HM-200 precision scale (A&D Engineering Inc., Bradford, Massachusetts, USA), and the mean reading was recorded.^13^^,^^15^^,^^16^

The samples were suspended by the nylon thread and placed two by two inside a plastic vessel with a wide opening containing 7.5 mL of deionized distilled water, taking care to avoid any contact between them and the inner surface of the container. The containers were sealed and left for 7 days in an incubator (37±1°C, 95±5% RH). Afterwards, the samples were removed from the containers, rinsed with deionized distilled water, blotted dry with absorbent paper, and placed in a dehumidifier for 24 hours. Then, the samples were weighed again, approaching the values to the nearest 0.001 g. The weight loss of each sample (initial mass minus final mass) was expressed as a percentage of the original mass (m% = mi – mf) and taken as the solubility of the sealer.^13^^,^^15^^,^^16^

### Radiopacity

As in Flores, et al.^14^ (2011) and Camargo, et al.^15^ (2017), to perform the radiopacity test, five acrylic plates (2.2 cm × 4.5 cm × 1 mm) containing wells measuring 1 mm in depth and 5 mm in diameter were prepared and placed over a glass plate covered by a cellophane sheet. Each well was filled with one of the sealers, following a sequence according to the setting time of the material so that the samples were ready for radiographic evaluation immediately after the final setting of all materials. To avoid the formation of bubbles, the freshly prepared sealer was introduced into the wells using a syringe. Another glass plate covered with cellophane was placed on top of the samples until complete setting, and any excess sealer was removed. Each plate was kept in an incubator (37±1°C, 95±5% RH) for a period corresponding to three times the sealer setting time.

At the time of radiographic exposure, each of the acrylic plates containing the sealers was positioned alongside another acrylic plate (1.3 cm × 4.5 cm × 1 mm) containing an aluminum step wedge made of 1100 alloy whose thickness ranged from 1 mm to 10 mm in uniform steps of 1 mm each (Margraf Dental MFG Inc., Jenkintown, Pennsylvania, USA). This set of acrylic plates was placed in front of a phosphor plate next to the aluminum step wedge, and a digital radiograph was taken using a Digora™ system (Soredex Orion Corporation, Helsinki, Finland). Radiographic images were obtained using a Spectro 70X X-ray machine (Dabi Atlante, Ribeirão Preto, São Paulo, Brazil) at 70 kVp and 8 mA. The object-to-focus distance was 30 cm, and the exposure time was 0.2 seconds. Exposed imaging plates of the test samples were immediately scanned after exposure (Soredex Orion Corporation, Helsinki, Finland) and analyzed using Digora™ for Windows, version 5.1.^14^^,^^15^

The Digora™ application software determines radiographic density (densitometric analysis) or, in other words, the RD of a given material through its gray levels (mm Al). Thus, a 2 mm^2^ area (44.5 × 44.5 px^2^) was standardized and used for each specimen in the radiographic images of the sealers.^14^^,^^15^

### Statistical analysis

The statistical analysis was performed to compare the effect of the activation protocols for each sealer. The results were preliminarily subjected to sample distribution (Shapiro-Wilk, *p*>0.05) and homogeneity of variance (Levene, *p*>0.05) tests. Solubility data were analyzed by the Kruskal-Wallis test, while setting time, flow, dimensional change, and radiopacity data were analyzed by one-way ANOVA. Both analyses were followed by Tukey's test for multiple comparisons. All tests were performed using SigmaPlot 11.0 with a 95% probability level (α=0.05).

## Results

Table 1 shows the results for the analysis of the physicochemical properties of root canal sealers according to the different activation protocols.

**Table 1:** Physicochemical properties of root canal sealers without activation, with ultrasonic activation, and with sonic activation protocols (mean±standard deviation)

Sealer	Activation protocol		Physicochemical property			
		Setting time (min)	Flow (mm)	Dimensional change (%)	Solubility (%)	Radiopacity (mmAl)
AH Plus	No activation	463.0 (1.45)^C^	34.48 (0.07)^C^	0.50 (0.36)^A^	0.73 (0.76)^AB^	7.65 (0.54)^B^
	Ultrasonic	991.33 (7.50)^A^	63.41 (0.23)^A^	2.30 (2.02)^A^	2.47 (0.63)^B^	9.20 (0.40)^A^
	Sonic	518.33 (14.57)^B^	38.60 (0.31)^B^	-3.85 (2.51)^B^	-0.51 (2.62)^A^	7.72 (2.72)^B^
MTA Fillapex	No activation	373.67 (137.71)^B^	55.33 (2.49)^C^	-5.40 (1.77)^A^	2.56 (3.64)^A^	3.04 (0.16)^B^
	Ultrasonic	1534.00 (26.51)^A^	72.74 (0.13)^A^	-4.96 (3.82)^A^	7.46 (9.77)^B^	2.85 (0.24)^B^
	Sonic	444.67 (40.22)^B^	59.45 (0.16)^B^	-13.73 (3.82)^B^	9.50 (0.88)^B^	4.83 (0.75)^A^
ADSeal	No activation	241.33 (9.71)^A^	55.16 (0.01)^C^	8.84 (4.05)^A^	-1.68 (1.96)^A^	4.34 (0.67)^A^
	Ultrasonic	142.00 (7.55)^B^	70.70 (0.16)^A^	6.07 (1.47)^A^	0.48 (6.62)^A^	3.08 (0.22)^B^
	Sonic	156.33 (6.11)^B^	58.34 (0.50)^B^	-2.72 (2.62)^B^	-3.64 (1.15)^A^	5.16 (1.05)^A^
GuttaFlow BioSeal	No activation	25.33 (1.53)^C^	34.43 (0.28)^C^	3.23 (5.03)^A^	-0.75 (1.01)^AB^	7.44 (0.61)^A^
	Ultrasonic	46.8 (1.53)^A^	55.08 (0.51)^A^	1.78 (3.34)^A^	-2.99 (4.20)^A^	7.44 (0.53)^A^
	Sonic	30.67 (2.08)^B^	41.05 (0.08)^B^	-1.97 (2.47)^A^	2.07 (1.08)^B^	5.33 (0.80)^B^
GuttaFlow 2	No activation	25.33 (1.15)^C^	33.72 (0.33)^C^	6.86 (5.31)^A^	-1.13 (0.88)^A^	7.03 (0.35)^B^
	Ultrasonic	46.00 (3.60)^A^	54.63 (0.01)^A^	-0.56 (0.49)^B^	0.20 (1.85)^A^	8.00 (0.17)^A^
	Sonic	33.00 (1.00)^B^	41.35 (0.38)^B^	-4.47 (4.68)^B^	-0.16 (1.77)^A^	6.31 (1.46)^B^

Uppercase different letters indicate statistically differences in column (between activation protocols) for each sealer (p<0.05)

### Setting time

Both ultrasonic and sonic activation increased the setting time of AHPlus, GuttaFlow Bioseal, and GuttaFlow 2, wherein ultrasonic activation had the highest values (*p*<0.05). The setting time of MTA Fillapex increased only after ultrasonic activation (*p*<0.05). As for the ADSeal sealer, the setting time decreased after ultrasonic and sonic activation, which were similar to each other.

### Flow

The flow (mm) was higher after ultrasonic activation, while sonic activation presented intermediate results for all tested sealers (*p*<0.05).

### Dimensional change

Sonic activation significantly reduced the percent values of dimensional change for AHPlus, MTA Fillapex, and ADSeal sealers (*p*<0.05), wherein ultrasonic activation did not significantly influence the dimensional change. For GuttaFlow 2, ultrasonic and sonic activation reduced the percentage of dimensional change (*p*<0.05).

### Solubility

AHPlus showed higher solubility when ultrasonically activated and lower solubility when sonically activated, and the NA group was at times similar to the sonic group and other times similar to the ultrasonic group. GuttaFlow Bioseal presented the lowest solubility when ultrasonically activated and the highest values when sonically activated, whereas the NA group was at times similar to the sonically activated group and other times similar to the ultrasonically activated group. MTA Fillapex showed higher solubility after ultrasonic and sonic activation when compared to the NA group (*p*<0.05).

### Radiopacity

Ultrasonic activation increased the radiopacity (mm Al) of AH Plus and GuttaFlow 2 sealers, and reduced the radiopacity of ADSeal. Sonic activation significantly increased the radiopacity of MTA Fillapex, and reduced the radiopacity of GuttaFlow Bioseal (*p*<0.05). The results show that all sealers in all activation protocols had radiopacity greater than 3 mm Al.

## Discussion

Sonic and ultrasonic activation devices are widely used to increase the effectiveness of irrigating solutions;^10^^,^^17^ however, studies on the effect of these devices on endodontic pastes and sealers are still recent and limited, demonstrating that sonic and ultrasonic activations are capable of increasing the penetration of endodontic sealers into dentinal tubules and improving the adaptation between the filling material and the dentin.^1^^,^^6^^,^^10^ However, it is still unclear what sonic and ultrasonic activation entails in the chemical reaction of different sealers and the impact on their physicochemical properties. Therefore, the methodology used to assess the effect of sonic and ultrasonic activation protocols on the physicochemical properties of the sealers was based on ANSI/ADA specification No. 57,^12^ with the modifications proposed by Carvalho-Júnior, et al.^16^ (2007).

The results of this study show that ultrasonic and sonic activation protocols alter all of the investigated physicochemical properties. Regarding ultrasonic activation, these changes probably occur because the activation of solid-liquid systems causes permanent physicochemical changes in this mixture owing to cavitation, formation of liquid microflow, disruption into solids and, consequently, instability at the interface of the system.^18^ Cavitation is the formation of air bubbles close to the irregular surfaces of solid particles, which can grow during several cycles until they reach a critical diameter, which induces their implosion.^6^^,^^19^ This collapse leads to extreme local conditions, such as very high pressure and temperatures, which directly interfere with the structure of the compounds,^19^ possibly producing variations, from organic decomposition to changes in the morphology of crystalline inorganic structures.^18^

The effects of sonic activation on the polymerization of sealers are less intense, leading to only physical changes due to the compression and rarefaction caused by the propagation of sound waves in the medium. Unlike ultrasonic activation, the sound wave does not drag the particles of the mixture, but only causes them to vibrate around their equilibrium position, causing hydrodynamic agitation and producing a small variation in temperature.^6^^,^^10^^,^^14^^,^^20^^–^^25^ Thus, depending on the molecular structure of the compounds, these effects may vary, which explains the diverse behavior of the different sealers evaluated in this study.

The setting time of AHPlus, GuttaFlow 2, and GuttaFlow BioSeal sealers was increased by both activation protocols, especially by ultrasonic activation. Moreover, ultrasonic activation significantly increased the setting time of MTA Fillapex. The use of ultrasonic devices associated with inserts that act at high frequency (25-40 kHz) induces turbulent flow in endodontic sealers and the formation of cavitation bubbles, increasing the temperature and pressure of the system,^6^^,^^26^^–^^28^ possibly generating radicals in the organic portion, making it responsible for the polymerization reaction, slowing it down. On the other hand, sonic activation operates through low-frequency vibrations (up to 10 kHz), which, by combining short movements inside and outside the root canal, synergistically create a hydrodynamic phenomenon.^26^^,^^29^ Although this hydrodynamic effect generated in endodontic sealers had low frequency and intensity, it may have been responsible for the increase in the temperature of the sealers, since sonic and ultrasonic activation can raise the temperature inside the root canals by up to 2°C,^14^^,^^20^^–^^25^ which would be enough to alter the physical properties of the mixture, directly influencing the setting time as well as the rheological properties of the tested sealers. It should be noted that only AHPlus and GuttaFlow 2 without activation met the ANSI/ADA standards, since the values obtained did not exceed 10% of what is determined by the manufacturer.

Only ADSeal showed an opposite behavior to that of the other tested sealers, since both activation protocols caused a decrease in setting time, even though it is an epoxy resin cement similar to AHPlus. There are few reports in the literature on the physicochemical properties of ADSeal.^30^^,^^31^ According to Marciano, et al.^32^ (2004) ADSeal presents a lower setting time compared to AH Plus, similar to the present study, which may be related to the different percentages and types of polymerizing agents (catalysts) present in the composition of these sealers,^32^ which, after ultrasonic activation, followed reaction mechanisms and eventually increased the speed of the polymerization reaction. Epoxy resin is a monomer which, when combined with polymerizing agents, initiates the polymerization process, wherein the type and amount of these agents can determine differences in the setting time of the resin.^31^

According to Ørstavik^20^ (1983), flowability can be influenced by setting time; however, although the activation protocols reduced the setting time of ADSeal, this property did not interfere with its flowability.^31^ The results of the present study show that after the sonic and ultrasonic activation protocols there was an increase in the flow values of all investigated sealers, for which ultrasonic activation had the highest flow values. The activation of endodontic sealers at high frequency and with a small amplitude of oscillations gives the sealers enough energy for the polymerization to occur in a more homogeneous way, providing better incorporation of the fill/loading particles of the organic matrix.^6^^,^^27^ In addition, note that the heat generated during this process reduces sealer viscosity, increasing its flow and the rheological and mechanical properties of the material, especially its cohesive strength.^6^^,^^19^^,^^27^^,^^33^ These characteristics, added to the increase in sealer pressure against the root canal walls, allow the filling of irregularities and recesses of the canal, greater penetration into accessory canals, isthmus, and dentinal tubules, with formation of a greater number, density, and extension of tags.^1^^,^^6^ Despite the flow changes caused by the activation protocols, all values found were greater than 20 mm in diameter, as determined by ANSI/ADA. However, note that excessive flow can increase the risk of apical extrusion and consequent postoperative pain.

The dimensional change test showed lower values after sonic activation of AHPlus, MTA Fillapex, ADSeal, and GuttaFlow 2. It is suggested that the hydrodynamic effect of sonic activation on the endodontic sealers and the consequent higher incorporation of the filling particles favored the packing of the molecular structures, altering the catalytic process of sealers, and consequently the levels of dimensional change. However, ultrasonic activation was able to reduce the dimensional change values only of GuttaFlow 2, whereas GuttaFlow BioSeal did not have its values influenced by the activation protocols. This difference between the two silicon-based sealers may be due to the incorporation of silver nanoparticles in the composition of GuttaFlow Bioseal, which requires the presence of stabilizers, preventing the aggregation of nanoparticles within the sealer.^35^ Thus, according to ANSI/ADA specification No. 57, which proposes that no material should exhibit contraction or dilation of more than 1% or 0.1%, respectively, the tested sealers were not in accordance with the proposed standards.

Ultrasonic and sonic activation did not interfere with the solubility of ADSeal and GuttaFlow 2 sealers. On the other hand, after the activation protocols, MTA Fillapex showed high solubility values, unlike AH Plus and GuttaFlow Bioseal, which did not follow the pattern of results, presenting higher or lower values depending on the activation protocol. These results are in line with previous studies that demonstrated the high solubility of MTA Fillapex when compared to other sealers.^34^^–^^38^ This characteristic is related to the process of alkalinization of the calcium hydroxide present in its composition, which leads to a greater release of Ca^2+^ ions in aqueous medium.^35^^,^^37^ Note that this process can also lead to an increase in sealer setting time.^38^ A recent literature review by Jafari and Jafari^39^ (2017) has stressed that, despite the good performance of MTA Fillapex regarding the release of calcium ions and flow, its high solubility remains a problem, which should be considered at the moment of clinical indication, since the dissolution of endodontic sealers can release irritants into periapical tissues, allowing the formation of gaps between root canals and the filling mass, making it susceptible to bacterial infiltration over time.^14^ Considering these results, only MTA Fillapex, regardless of the activation protocol, does not comply with ANSI/ADA standards.

The radiopacity test shows variations in the behavior of sealers towards the activation protocols. This variation can be related to the ultrasonic cavitation phenomenon, which induces the implosion of air bubbles and causes locally extreme temperature and pressure conditions, in combination with micro-streaming generated by cavitation oscillations, leading to dispersion effects and splitting up of particle agglomerates^18^^,^^19^ such as the inorganic components present in each of the evaluated sealers, possibly leading to morphological transformation of the structures of radiopacifying agents and the formation of crystals with completely different unit cells. Thus, it can be proposed that the varied behavior observed in radiopacity was altered as a function of the changes in the crystalline structures of the radiopacifying agents. As for sonic activation, variation in radiopacity may be related to a higher or lower exposure to the inorganic compounds present, which occurred randomly due to the hydrodynamic motion caused by sound waves.^6^^,^^10^

However, all of the investigated sealers, regardless of the activation protocol, presented radiographic density higher than 3 mm Al as proposed by ANSI/ADA specification No. 57.^13^^,^^14^^,^^16^^,^^40^ The radiographic images of the sealers were obtained with use of a Digora™ digital system using a sensor and replacing the conventional radiographic film, and using Digora™ for Windows, version 1.51, which allowed for a more accurate capture, processing, storage and measurement of the results when compared to the conventional radiographic film analysis, in addition to the need for low exposure for the sensor sensitization.^15^^,^^16^^,^^40^

## Conclusions

It is known that the activation of endodontic sealers improves root canal sealing, especially in areas of difficult access such as lateral and accessory canals, isthmus, recesses, and apical deltas.^1^^,^^6^ However, this study allowed to conclude that directly tested sonic and ultrasonic activation altered the physicochemical properties of endodontic sealers. In this regard, it is important that, prior to clinical application, the physicochemical properties of sealers associated with activation techniques be evaluated in order to determine whether the properties are maintained within the parameters set by ANSI/ADA, ensuring safety and quality of endodontic filling. Moreover, it is important that the dental practitioner consider the advantages and disadvantages of employing activation in each specific clinical case.
